# Supplementary material for: An optimized messenger RNA vaccine candidate protects non-human primates from Zika virus infection
Source: NPJ Vaccines. 2023 Apr 20;8:58. doi: 10.1038/s41541-023-00656-4 (PMC10119314; doi:10.1038/s41541-023-00656-4)
Supplement: Supplementary file 1 — Supplemental Material [file 41541_2023_656_MOESM1_ESM.pdf]

Supplementary Information

Supplementary Table 1. Summary of statistical comparisons between nAb titers measured at week 4 (day 28) and week 8 (day 56) in NHPs

**Comparisons**	**Adjusted *P* Values**	
	**Week 4**	**Week 8**
Control, 200 µg x2 vs. mRNA-1325, 10 µg x2	0.0059	<0.0001
Control, 200 µg x2 vs. mRNA-1325, 50 µg x2	0.0017	0.0004
Control, 200 µg x2 vs. mRNA-1325, 200 µg x1	0.0004	0.0025
Control, 200 µg x2 vs. mRNA-1325, 200 µg x2	0.0002	<0.0001
Control, 200 µg x2 vs. mRNA-1893, 10 µg x2	0.0024	<0.0001
mRNA-1325, 10 µg x2 vs. mRNA-1325, 50 µg x2	0.3309	0.4673
mRNA-1325, 10 µg x2 vs. mRNA-1325, 200 µg x1	0.0214	0.9571
mRNA-1325, 10 µg x2 vs. mRNA-1325, 200 µg x2	0.0511	0.0007
mRNA-1325, 10 µg x2 vs. mRNA-1893, 10 µg x2	0.0851	0.0459
mRNA-1325, 50 µg x2 vs. mRNA-1325, 200 µg x1	0.4120	0.2916
mRNA-1325, 50 µg x2 vs. mRNA-1325, 200 µg x2	0.9795	0.4697
mRNA-1325, 50 µg x2 vs. mRNA-1893, 10 µg x2	0.8869	0.9600
mRNA-1325, 200 µg x1 vs. mRNA-1325, 200 µg x2	0.8678	0.0379
mRNA-1325, 200 µg x1 vs. mRNA-1893, 10 µg x2	0.9995	0.0568
mRNA-1325, 200 µg x2 vs. mRNA-1893, 10 µg x2	>0.9999	0.9719

nAb, neutralizing antibody; NHP, non-human primate.

Supplementary Table 2. Summary of statistical comparisons between nAb titers measured on day 56 in mice

Comparisons	Adjusted *P* Values
2+1µg:mRNA-1893+Control vs. 2+1µg:CprME+NS2B3	0.9988
2+1µg:mRNA-1893+Control vs. 2+1µg:C2AprME+Control	0.9810
2+1µg:mRNA-1893+Control vs. 0.4+0.2µg:mRNA-1893+Control	0.0036
2+1µg:mRNA-1893+Control vs. 0.4+0.2µg:CprME+NS2B3	0.6693
2+1µg:mRNA-1893+Control vs. 0.4+0.2µg:C2AprME+Control	<0.0001
2+1µg:CprME+NS2B3 vs. 2+1µg:C2AprME+Control	0.4435
2+1µg:CprME+NS2B3 vs. 0.4+0.2µg:mRNA-1893+Control	0.0002
2+1µg:CprME+NS2B3 vs. 0.4+0.2µg:CprME+NS2B3	0.1155
2+1µg:CprME+NS2B3 vs. 0.4+0.2µg:C2AprME+Control	<0.0001
2+1µg:C2AprME+Control vs. 0.4+0.2µg:mRNA-1893+Control	0.1098
2+1µg:C2AprME+Control vs. 0.4+0.2µg:CprME+NS2B3	>0.9999
2+1µg:C2AprME+Control vs. 0.4+0.2µg:C2AprME+Control	0.0006
0.4+0.2µg:mRNA-1893+Control vs. 0.4+0.2µg:CprME+NS2B3	0.4283
0.4+0.2µg:mRNA-1893+Control vs. 0.4+0.2µg:C2AprME+Control	0.7084
0.4+0.2µg:CprME+NS2B3 vs. 0.4+0.2µg:C2AprME+Control	0.0043

CprME, capsid, premembrane/membrane, and envelope; nAb, neutralizing antibody.

Supplementary Figure. Uncropped Blots with Molecular Weight Markers

Figure 1A

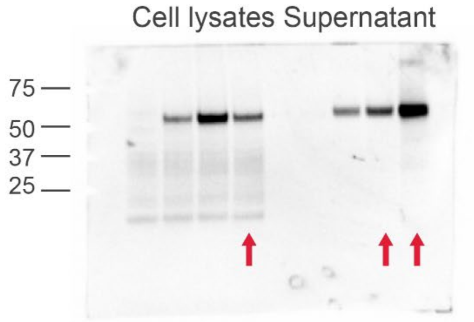

Red arrows indicate irrelevant lanes not included in figure. (These were additional ZIKV mRNA constructs that did not advance farther.)

Figure 1B

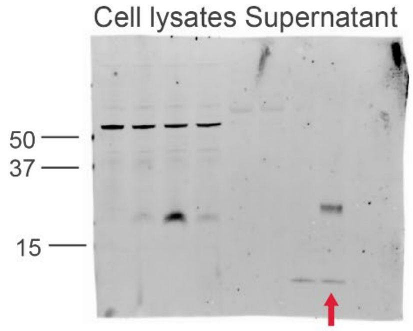

Figure 2A

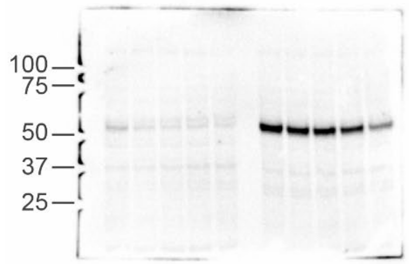

Figure 2B

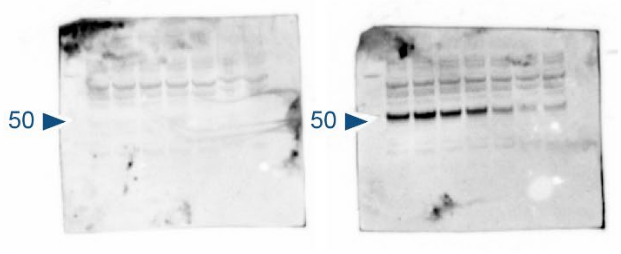

Figure 2C

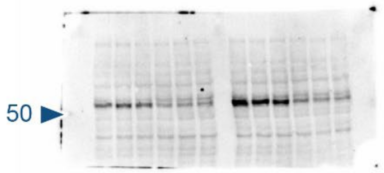

Figure 2D

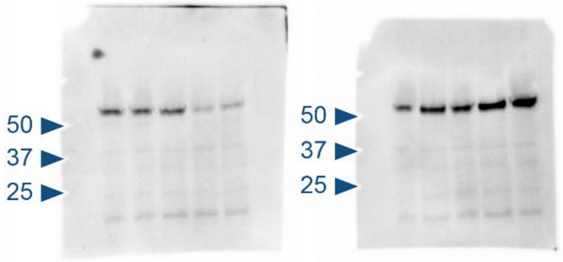

Figure 3B

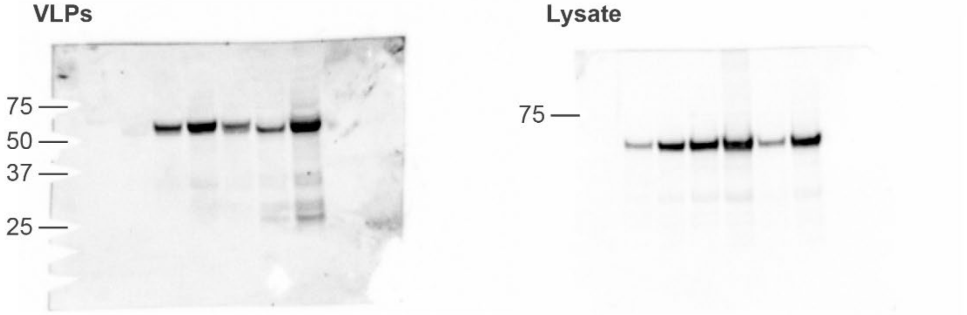

Figure 6A top panel

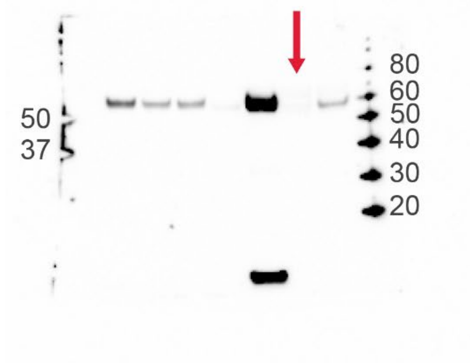

Figure 6B top panel

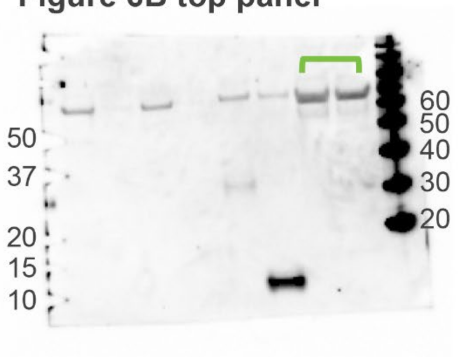

Figure 6A bottom panel

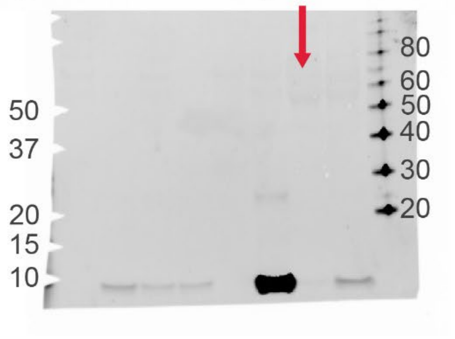

Figure 6B bottom panel

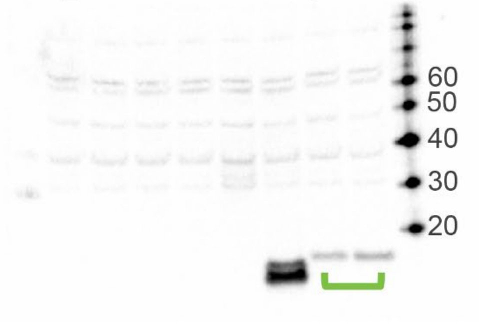

6A gels: red arrow indicates a skipped well.

6B gels: green bracket indicates same sample run in duplicate.
