# Supplementary material for: An optimized messenger RNA vaccine candidate protects non-human primates from Zika virus infection
Source: NPJ Vaccines. 2023 Apr 20;8:58. doi: 10.1038/s41541-023-00656-4 (PMC10119314; doi:10.1038/s41541-023-00656-4)
Supplement: Supplementary file 2 — REPORTING SUMMARY [file 41541_2023_656_MOESM2_ESM.pdf]

nature portfolio

Corresponding author(s): Brooke Bollman

Last updated by author(s): Sep 16, 2022

Reporting Summary

Nature Portfolio wishes to improve the reproducibility of the work that we publish. This form provides structure for consistency and transparency in reporting. For further information on Nature Portfolio policies, see our Editorial Policies and the Editorial Policy Checklist.

Statistics

For all statistical analyses, confirm that the following items are present in the figure legend, table legend, main text, or Methods section.

n/a	Confirmed	
☐	☒	The exact sample size (n) for each experimental group/condition, given as a discrete number and unit of measurement
☐	☒	A statement on whether measurements were taken from distinct samples or whether the same sample was measured repeatedly
☐	☒	The statistical test(s) used AND whether they are one- or two-sided<br>*Only common tests should be described solely by name; describe more complex techniques in the Methods section.*
☒	☐	A description of all covariates tested
☐	☒	A description of any assumptions or corrections, such as tests of normality and adjustment for multiple comparisons
☐	☒	A full description of the statistical parameters including central tendency (e.g. means) or other basic estimates (e.g. regression coefficient) AND variation (e.g. standard deviation) or associated estimates of uncertainty (e.g. confidence intervals)
☐	☒	For null hypothesis testing, the test statistic (e.g. F, t, r) with confidence intervals, effect sizes, degrees of freedom and P value noted<br>*Give P values as exact values whenever suitable.*
☒	☐	For Bayesian analysis, information on the choice of priors and Markov chain Monte Carlo settings
☒	☐	For hierarchical and complex designs, identification of the appropriate level for tests and full reporting of outcomes
☒	☐	Estimates of effect sizes (e.g. Cohen's d, Pearson's r), indicating how they were calculated

Our web collection on statistics for biologists contains articles on many of the points above.

Software and code

Policy information about availability of computer code

Data collection	Attune NxT Software v4.2.0; Pherastar FSX Software v5.70 R4
Data analysis	Microsoft Excel; FlowJo v10.8.0; GraphPad Prism 9

For manuscripts utilizing custom algorithms or software that are central to the research but not yet described in published literature, software must be made available to editors and reviewers. We strongly encourage code deposition in a community repository (e.g. GitHub). See the Nature Portfolio guidelines for submitting code & software for further information.

Data

Policy information about availability of data

All manuscripts must include a data availability statement. This statement should provide the following information, where applicable:

- Accession codes, unique identifiers, or web links for publicly available datasets
- A description of any restrictions on data availability
- For clinical datasets or third party data, please ensure that the statement adheres to our policy

Upon request, and subject to review, Moderna, Inc. will provide the data that support the findings of this study.

Field-specific reporting

Please select the one below that is the best fit for your research. If you are not sure, read the appropriate sections before making your selection.

☒ Life sciences ☐ Behavioural & social sciences ☐ Ecological, evolutionary & environmental sciences

For a reference copy of the document with all sections, see nature.com/documents/nr-reporting-summary-flat.pdf

Life sciences study design

All studies must disclose on these points even when the disclosure is negative.

Sample size	Sample sizes were determined based on the criteria set by the institutional Animal Care and Use Committee.
Data exclusions	No data were excluded from analyses.
Replication	Figure 1 and Figure 6: Data are representative of a single experiment, with error bars indicating the SD of technical repeats on the same plate (ie, triplicate wells).
Randomization	NA
Blinding	Investigators were not blinded, as this was a preclinical study.

Reporting for specific materials, systems and methods

We require information from authors about some types of materials, experimental systems and methods used in many studies. Here, indicate whether each material, system or method listed is relevant to your study. If you are not sure if a list item applies to your research, read the appropriate section before selecting a response.

Materials & experimental systems

n/a	Involved in the study	
☐	☒	Antibodies
☐	☒	Eukaryotic cell lines
☒	☐	Palaeontology and archaeology
☐	☒	Animals and other organisms
☒	☐	Human research participants
☒	☐	Clinical data
☒	☐	Dual use research of concern

Methods

n/a	Involved in the study	
☒	☐	ChIP-seq
☐	☒	Flow cytometry
☒	☐	MRI-based neuroimaging

Antibodies

Antibodies used	Human monoclonal antibodies (mAbs) ZV-116 and ZV-117 were provided by Dr. James Crowe (Vanderbilt University Medical Center, Nashville, TN). Mouse mAb ZV-67 was provided by Dr. Michael Diamond (Washington University School of Medicine, St. Louis, MO). Commercially available flavivirus E protein-specific mAb 4G2 (EMD Millipore, Darmstadt, Germany), rabbit anti-ZIKV prM and C antibodies (GeneTex, Irvine, CA), and goat horseradish peroxidase (HRP)-conjugated anti-human, anti-mouse, and anti-rabbit immunoglobulin G secondary antibodies (Southern Biotech, Birmingham, AL) were used.
Validation	ZV-117: https://www.nature.com/articles/nature20564<br>ZV-67: https://www.cell.com/fulltext/S0092-8674(16)30927-8<br>4G2: https://www.emdmillipore.com/US/en/product/Anti-Flavivirus-Group-Antigen-Antibody-clone-D1-4G2-4-15,MM_NF-MAB10216<br>anti-ZIKV prM: https://www.genetex.com/Product/Detail/Zika-virus-prM-protein-antibody/GTX133305<br>anti-ZIKV C: https://www.genetex.com/Product/Detail/Zika-virus-Capsid-protein-antibody/GTX133317

Eukaryotic cell lines

Policy information about cell lines

Cell line source(s)	293T and Vero cells were obtained from the cell bank (Moderna, Inc.)
Authentication	Cell lines from Moderna's cell bank were established from an initial purchase from ATCC (Catalog numbers: CRL-3216 [293T]; CCL-81 [Vero]). Short tandem repeat (STR) profiling was performed by ATCC for 293T cells.
Mycoplasma contamination	Cell lines were tested routinely every quarter and confirmed to be free from mycoplasma contamination.

Commonly misidentified lines (See ICLAC register)	No commonly misidentified lines were used in this study.

Animals and other organisms

Policy information about studies involving animals; ARRIVE guidelines recommended for reporting animal research

Laboratory animals	Mice: C57BL/6 mice (Charles River Laboratories [Wilmington, MA]) Naïve Indian-origin rhesus macaques: National Institutes of Health/Vaccine Research Center-owned animals, holding location Bioqual Inc.
Wild animals	The study did not involve wild animals
Field-collected samples	The study did not involve samples collected from the field
Ethics oversight	Experiments involving animals were carried out in compliance with approval from the Animal Care and Use Committee of Moderna, Inc.

Note that full information on the approval of the study protocol must also be provided in the manuscript.

Flow Cytometry

Plots

Confirm that:

- [ ] The axis labels state the marker and fluorochrome used (e.g. CD4-FITC).
- [ ] The axis scales are clearly visible. Include numbers along axes only for bottom left plot of group (a 'group' is an analysis of identical markers).
- [ ] All plots are contour plots with outliers or pseudocolor plots.
- [ ] A numerical value for number of cells or percentage (with statistics) is provided.

Methodology

Sample preparation	Flow cytometry was used for all experiments that employed the GFP reporter virus.
Instrument	NA
Software	NA
Cell population abundance	NA
Gating strategy	NA

- [ ] Tick this box to confirm that a figure exemplifying the gating strategy is provided in the Supplementary Information.
